# Enhancing the Concentration Capability of Nonsupported Electrically Driven Liquid-Phase Microextraction through Programmable Flow Using an All-In-One 3D-Printed Optosensor: A Proof of Concept

**DOI:** 10.1021/acs.analchem.4c02139

**Published:** 2024-06-25

**Authors:** Ali Sahragard, Enrique Javier Carrasco-Correa, David J Cocovi-Solberg, Pavel Kubáň, Manuel Miró

**Affiliations:** † FI-TRACE Group, Department of Chemistry, Faculty of Science, University of the Balearic Islands, Carretera de Valldemossa km 7.5, E-07122 Palma de Mallorca, Illes Balears, Spain; ‡ CLECEM Group, Department of Analytical Chemistry, University of Valencia, C/Doctor Moliner, 50, 46100 Burjassot, Valencia, Spain; § Department of Chemistry, Institute of Analytical Chemistry, University of Natural Resources and Life Sciences Vienna, Muthgasse 18, 1190 Vienna, Austria; ∥ Institute of Analytical Chemistry of the Czech Academy of Sciences, Veveří 97, CZ-60200 Brno, Czech Republic

## Abstract

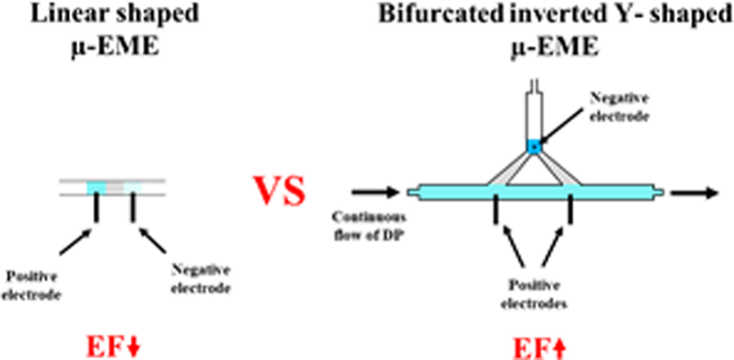

A versatile millifluidic 3D-printed inverted Y-shaped unit (3D-YSU) was prototyped to ameliorate the concentration capability of nonsupported microelectromembrane extraction (μ-EME), exploiting optosensing detection for real-time monitoring of the enriched acceptor phase (AP). Continuous forward-flow and stop-and-go flow modes of the donor phase (DP) were implemented via an automatic programmable-flow system to disrupt the electrical double layer generated at the DP/organic phase (OP) interface while replenishing the potentially depleted layers of analyte in DP. To further improve the enrichment factor (EF), the organic holding section of the OP/AP channel was bifurcated to increase the interfacial contact area between the DP and the OP. Exploiting the synergistic assets of (i) the continuous forward-flow of DP (1050 μL), (ii) the unique 3D-printed cone-shaped pentagon cross-sectional geometry of the OP/AP channel, (iii) the bifurcation of the OP that creates an inverted Y-shape configuration, and (iv) the in situ optosensing of the AP, a ca. 24 EF was obtained for a 20 min extraction using methylene blue (MB) as a model analyte. The 3D-YSU was leveraged for the unsupervised μ-EME and the determination of MB in textile dye and urban wastewater samples, with relative recoveries ≥88%. This is the first work toward analyte preconcentration in μ-EME with in situ optosensing of the resulting extracts using 3D-printed millifluidic platforms.

Electromembrane extraction (EME) has been extensively exploited over the past decade to extract charged analytes, most often ionizable drugs or small-molecular-mass biomolecules, from biological and environmental samples.^1−7^ Three-phase EME capitalizes on the exertion of an electrical driving force to enable the selective transfer of charged analytes from the sample (donor phase (DP)) through an immiscible organic phase (OP) that acts as the insulating barrier, usually embedded in a hydrophobic porous membrane (so-called supported EME), into the aqueous acceptor phase (AP).^3^ Several new formats, configurations, and extraction phases for EME have been introduced as of yet, namely, nanomaterial-assisted supported EME, gel-based supported EME, and EME with biodegradable membranes or green solvents (e.g., neoteric solvents), just to name a few.^3,8−12^ Notwithstanding the widespread use of supported EME systems, some challenges still have to be faced to ameliorate the method’s repeatability because of the inability to precisely determine volumes of organic solvents embedded in the supporting phase and the nonuniformity of the polymer pores.^13^ In the meantime, there has been an increasing interest in accommodating supported EME in micro/millifluidic platforms with subsequent off-line/at-line liquid chromatography (LC)- or capillary electrophoresis (CE)-UV/mass spectrometric detection,^15,16^ and in few instances with online detection.^14,15^ The main issue of fluidic platforms accommodating supported EME that precludes method automation is the need for continuous regeneration of the liquid membrane because of the progressive washing out of the OP or the irreversible extraction of organic interfering compounds, whereupon the chip must be opened and the membrane replaced manually after every individual assay or sample batch.^16,17^ To tackle this shortcoming, the concept of nonsupported micro-EME (μ-EME) with automatic regeneration of the organic phase by programmable flow in every single extraction without human intervention proved to be a superb alternative.^18,19^ Unlike supported liquid membrane (SLM)-based EME, a plug of organic solvent is sandwiched between the DP and AP in μ-EME, mostly using perfluoroalkoxy and polytetrafluoroethylene (PTFE) tubings or pipet tips.^19,20^ In μ-EME, phase formation can be readily automated with flow analysis so that varied and precise volumes can be used at will while increasing the stability and reliability with regard to the electrical current across EME and the extraction efficiency in complex real samples.^13,18^ Despite the aforementioned advantages, single-line μ-EME bears significantly lower surface-to-volume ratios than those of standard SLMs; additionally, because the sample in μ-EME is stagnant, poor to moderate enrichment factors (EFs) are usually reported for μ-EMEs. Transfer of analytes might also potentially be hindered due to the accumulation of ions at the DP/OP and OP/AP interfaces, thereby resulting in reduced μ-EME recoveries and EFs of target ionizable analytes.^13,18^ Therefore, there is a quest for designing and manufacturing novel fluidic platforms with intricate channel configurations enabling flexible handling of the DP, OP, and AP.

Micromilling, a computer-controlled microcarving technology, has been vastly exploited to create milli/mesoanalytical devices with channels and reservoirs for flow-based supported EME applications.^21−23^ As opposed to its microfluidic counterparts fabricated by subtractive manufacturing, investment in clean room facilities or usage of sophisticated instruments is not needed, yet its applicability remains restricted to hard substrate materials, such as poly(methyl methacrylate) (PMMA),^24^ that bear limited chemical compatibility. Additionally, the elevated temperature generated during carving might pose deformational problems to the channels.^25^ Alignment difficulties and possible breakage of micromills might also jeopardize the ease of fabrication and device-to-device reproducibility.^25,26^ As an appealing alternative, 3D-printing technology, a subset of additive manufacturing processes, has recently been embraced in analytical chemistry on account of the increasing availability of printable materials and the inherent capabilities for creating bespoke geometries and complex designs of modules, scaffolds, housing, and accessories.^27−33^ In sample preparation applications, 3D-printing technology has been primarily utilized to fabricate platforms catering to solid-phase (micro)extraction^34−36^ and to a lesser extent to accommodating the various extraction modes of liquid-phase microextraction (LPME).^28,37^ In fact, 3D-printing in LPME workflows, especially EME, has not yet been leveraged to its full potential to outperform standard protocols for fluidic supported membrane-based EME and nonsupported μ-EME approaches that are both characterized by rigid architectures.

In an effort to address the challenges of limited EFs of μ-EME protocols, especially for low log *P* analytes, a novel approach based on low force stereolithography is herein proposed for manufacturing fluidic 3D-printed inverted T-shaped units (3D-TSUs) with diverse functionalities. This strategy is aimed at increasing (i) the volume of DP, (ii) the contact times, and (iii) the transfer area across the OP in an automated mode while monitoring the enriched AP through optical-fiber-based optosensing for the first time. The proposed configuration enables dynamic movement of the DP in a programmable flow-through mode, while the OP and AP remain stagnant. To maximize the analytical performance of 3D-TSU-μ-EME, different cross-sectional geometries of the OP/AP channel, including triangle, square, pentagon, circle, and obround shapes, are explored. Additionally, bifurcation of the OP channel is implemented to further enhance the contact area between OP and DP. The proof-of-concept applicability of the novel bifurcated 3D-printed inverted Y-shaped millifluidic unit (3D-YSU) accommodating automatic μ-EME is leveraged for matrix cleanup, preconcentration, and determination of methylene blue (MB) as a model of a highly polar analyte in untreated synthetic textile dye and urban wastewater samples at realistic MB concentrations.

## Materials and Methods

### Chemicals and Standards

See details in the Supporting Information.

### Fabrication of the Millifluidic Units

The details of the fabrication of the 3D-printed units are provided in the Supporting Information. Three 3D-printed prototypes with distinct functionalities were manufactured. Device 1 (V1) (Figure S1I) shared an external cubic geometry that was 25.5 mm wide, 32.5 mm high, and 13.5 mm deep. The molds for tapering threads were cylindrical, measuring 7 mm in height and 5 mm in diameter. The DP channel of V1 featured a circular cross-section with a length of 15.5 mm and a diameter of 2.5 mm. This channel was connected to two threaded holes acting as the sample inlet and outlet, respectively, through small channels featuring a length of 1.5 mm and a diameter of 1.0 mm each (see Figure S1I). The 10 mm long AP channel featured a 2.5 mm (length) × 2.5 mm (width) obround geometry based on our previous research.^38^ The AP channel was also connected to the threaded holes through a small channel of dimensions equal to those mentioned earlier for the DP channel. The holes for the two electrodes were cylindrically shaped, measuring 2 mm in depth and 2 mm in diameter, and were connected to the AP and DP channels, respectively.

In the V2 3D-TSU device (Figure S1II), the sizes of the channels, threads, and electrode holes were identical to those described in V1 except for the length of the AP channel, which was enlarged up to 15 mm. In addition, optical fiber holes were arranged in an upright position to the electrode in the AP. To this end, two ancillary holes with diameters of 3.4 mm and lengths of ca. 8 mm were designed for coupling the optical fibers to the 3D printed structure. The distance between the bottom of the optical fiber holes to the OP/AP channel was about 1.5 mm. Additionally, the AP channel featured varied cross-sectional geometries in V2, including triangle (4 mm high and 2.5 mm base), square (2.1 mm side), pentagon (1.65 mm side), circle (2.5 mm diameter), and obround (2.5 mm long and 2.5 mm wide) cross-sections (Figure S2). All of the designs maintain relatively similar volumes for an OP length of 3.5 mm.

**Figure 1:**
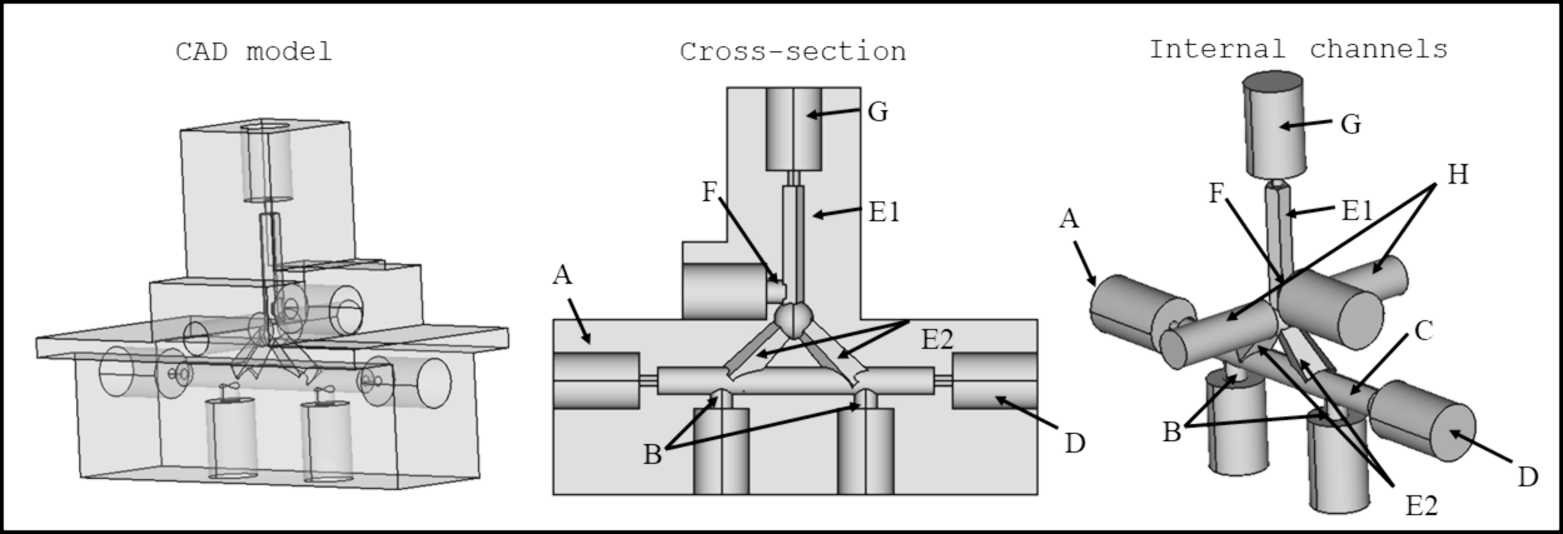
Design of the 3D-YSU V3 device with a bifurcated OP channel (E2) for the dynamic μ-EME of MB. (A) Thread for the DP input, (B) holes for the two positive electrodes in the DP channel, (C) DP channel, (D) thread for the DP output (waste), (E1) AP channel, (E2) OP bifurcated channels, (F) hole for the negative electrode in the AP channel, (G) thread for the OP/AP input, and (H) holes for the optical fibers.

In the V3 3D-YSU device (Figure 1), the sizes of the channels, threads, electrode holes, and optical fiber holders were kept equal to those for V2, but the cross-section of the AP channel was pentagon-shaped with a 1.25 mm side length and the OP holding part of the OP/AP channel was bifurcated to two new channels (see section Bifurcation of the OP Channel in Results and Discussion). In the V3 device, two positive electrodes were positioned in the DP close to the two OP-containing channels and one negative electrode was placed in the AP akin to V2. A total of 12 V1 3D-TSUs, 12 V2 3D-TSUs, or 8 V3 3D-YSUs could be 3D-printed at a time. The resin volumes for manufacturing one V1, V2, and V3 3D-TSU/YSU were 14.3, 13.4, and 12.8 mL, respectively.

### Flow System and Optical Detection

**Figure 2:**
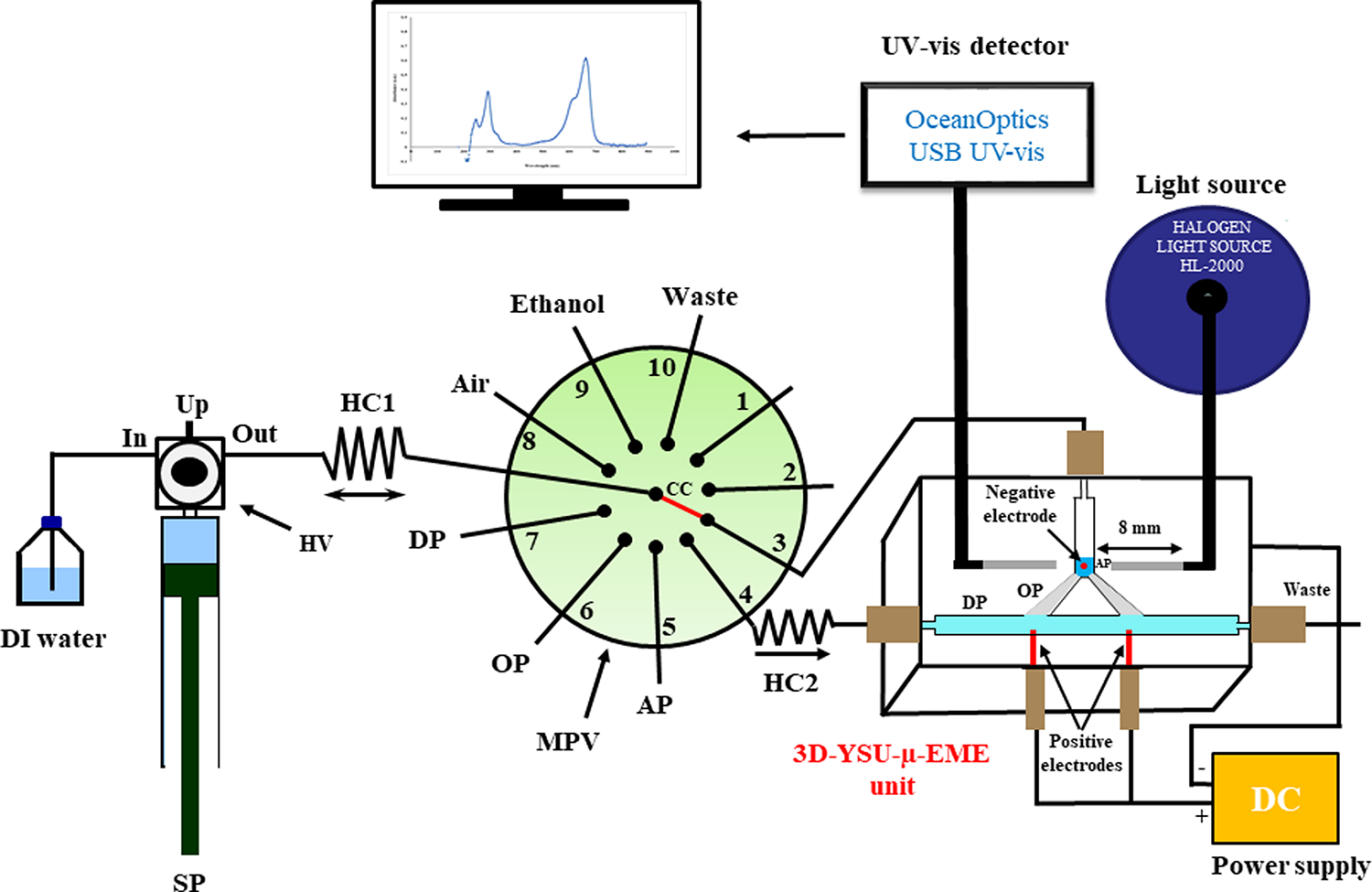
Schematic illustration of the SI-3D-YSU-μ-EME optosensing setup (V3 device) for automatic μ-EME and cleanup of MB-containing wastewaters. SP, syringe pump; HV, head valve; MPV, multiposition valve; HC1 and HC2, holding coils; CC, central channel; μ-EME, microelectromembrane extraction; DC, direct current power supply; DP, donor phase; OP, organic phase; AP, acceptor phase.

See the details in the Supporting Information and Figure 2.

### μ-EME Instrumentation

See the details in the Supporting Information.

### In-Line SI-μ-EME Procedure

The automatic μ-EME workflow starts by dispensing the DP toward the 3D-TSU/YSU device. To this end, 1050 μL of DP/sample (2 × 525 μL) was aspirated from port #7 of the multiposition valve (MPV) (see Figure 2) into holding coil 1 (HC1) and then dispensed to port #4 of MPV. The DP was introduced into HC2 (ca. 60 or 90 cm long PTFE tubing with 1.5 mm ID and 2.4 mm OD for 1050 or 1500 μL of DP, respectively) connected to the 3D-TSU/YSU prior to the OP/AP formation. In fact, the OP is usually partially displaced if the full DP is dispensed forward by the sequential injection (SI) system after OP formation. HC1 was then thoroughly washed consecutively with 200 μL of ethanol and 1000 μL of deionized (DI) water to eliminate remnants of MB on the HC1 walls. After dispensing the DP, the OP (24 μL in V1 and V2 and 110 μL in V3) and AP (44 μL in V1 and 10 μL in V2 and V3) were sequentially aspirated from their respective ports (#6 for the OP and #5 for the AP) and then brought to port #3. A plug of air (100 μL) was then introduced behind these phases to guide them to the designated position for μ-EME (see Figure 2). In the next step, the DP was pushed by a 50 μL plug of air to enable the electric contact and the stabilization of the DP/OP interface, whereupon the power supply was automatically activated and μ-EME was conducted at 250 V with a continuous forward-flow of the DP at 50 μL/min for a total extraction time of 20 min. After μ-EME, the CocoSoft freeware activated the SpectraSuite software for real-time recording of the spectra in the AP for V2 and V3 as detailed above. The operational procedure for in-line downstream spectrophotometric detection of the MB-laden AP after μ-EME in V1 has been reported elsewhere.^38^

After optical detection, HC1, HC2, and the 3D-TSU/YSU channels were washed with 300 μL of ethanol (100 μL for the DP channel, 100 μL for OP/AP channels, and 100 μL for the detection line connected to port #2 in V1, not shown in Figure 2) and 750 μL of water (250 μL for the DP channel, 250 μL for OP/AP channels, and 250 μL for the detection line connected to port #2 in V1, not shown in Figure 2) and flushed with 2300 μL of air (1800 μL for the DP channel, 250 μL for OP/AP channels, and 250 μL for the detection line connected to port #2) for V1; the channels were washed with 300 μL of ethanol (100 μL for the DP channel and 200 μL for OP/AP channels) and 500 μL of water (250 μL for the DP channel and 250 μL for OP/AP channels) and flushed with 2050 μL of air (1800 for the DP channel and 250 μL for OP/AP channels) for V2 and V3 (see Table S1). After this step, CocoSoft activated the next extraction run. For the stop-and-go experiments, the total volume was 300 μL, and the extraction time was 20 min in all experiments. Under each particular condition, the total volume was divided into time segments (see section Evaluation of Continuous Forward-Flow against Stop-and-Go Flow Modes of DP in Results and Discussion). To conduct the extraction processes, e.g., for 4 × 300 s, 75 μL of DP is first pumped into the DP channel, and then the flow is stopped for 300 s under the application of 250 V; this procedure is repeated four times.

### Real Samples

See the details in the Supporting Information.

### EF Calculation

See the details in the Supporting Information.

## Results and Discussion

The μ-EME conditions including the voltage (250 V), the pHs of the DP and AP, the OP length (3.5 mm), and the dimensions and the cross-sectional geometry of the OP/AP channel for the 3D-TSU V1 device were adopted from our previous study.^38^ As can be seen later in the section Bifurcation of the OP Channel for Further Enrichment, the electrical currents were lower than 1 μA throughout the extraction process. With such low currents, bubbles are negligibly generated and pose no stability concerns for the μ-EME performance. It is notable that 300 V was also investigated in this study, but it did not improve the EF despite the phases being stable during the entire μ-EME. A preliminary evaluation of the performance of the SI-μ-EME system with the V1 device was undertaken by connecting the outlet to a flow-through cell for downstream optical detection. However, the concentration capabilities of the V1 device were jeopardized as a consequence of several issues: (i) unreliable retrieval of the AP from the 3D-TSU toward HC1 and then forward dispensation to the UV–vis flow cell, (ii) potential sorption of MB to the PTFE tubing walls during backward- and forward-flows, and (iii) loss of a large volume of the AP during the retrieval and heart-cut injection to the flow cell (to avoid collection of OP), so a volume of ≥44 μL was required to record reliable UV–vis data. In fact, a preliminary comparison of the performance of the 3D-TSU V1 device connected to the flow cell (see section Flow System and Optical Detection) against *in situ* optosensing detection (V2 device) for a set of experimental conditions (extraction voltage of 250 V; extraction time of 20 min; OP of 1-octanol; 24 μL of OP; 44 μL of AP in V1 and 10 μL in V2; and 1050 μL of DP containing 4 mg/L MB) revealed an EF of ca. 0.3 versus that of ca. 4.6, indicating a larger concentration of MB in the close vicinity of the AP/OP interface in the time course of the μ-EME.

### Evaluation of μ-EME Parameters Using 3D-TSU with Optosensing Detection

Based on the preliminary results described above, further research was conducted for the selection of the experimental parameters of the automatic μ-EME system using the 3D-TSU V2 device, which enables real-time optosensing. The flow rate for handling the liquid plugs and generating the OP/AP interface was set here to 60 μL/min. Higher flow rates had detrimental effects on the phase formation process because a large amount of OP became scattered around the walls or across the T-connection, which was aligned with our previous findings indicating that flow rates >30 μL/min might jeopardize the reliability of the three (DP/OP/AP) plugs using 1-nonanol as the OP.^19^ The inability to use flow rates >30 μL/min using 1-nonanol could be associated with its higher viscosity compared to octanol. A constant voltage of 250 V was imposed for the μ-EME in all experiments according to our previous finding for nonsupported μ-EME of MB.^38^ Volumes of OP were investigated within the range of 18–30 μL. Experimental results indicated that 24 μL of OP that rendered a ca. 3.5 mm long plug, measured experimentally, avoided the collapse of the three-phase system while enduring high voltage and the dynamic flow-through DP mode. It should be noted that a minute volume of 1-octanol is lost through the formation of a wetting film throughout the connecting PTFE tubing walls,^19,39^ thus the experimental volume (24 μL) is larger than the theoretical value (ca. 18 μL). The lowest volume of OP fulfilling the stability requirements is here chosen to prevent high electrical resistance across the OP. Direct monitoring of the stagnant AP in the V2 device is feasible with the incorporated optosensing detection. This approach contrasts with our previous study^38^ and the V1 device in this work, in which the concentrated AP was diluted to fill the flow-through microcuvette. In V2, the AP volume was reduced as practically as possible, viz., 10 μL against 44 μL in V1, respectively, while ensuring the electrical connection and the reproducible phase formation throughout the 3D-TSU device.

In the optosensing 3D-TSU V2 configuration, the optical fibers were positioned to monitor the AP in the vicinity of the OP/AP interface (Figure S1II). It is important to mention that the optical fibers were kept ca. 8 mm away from the AP channel. In fact, too much light reached the detector at shorter distances and thus the method’s sensitivity and detectability based on absorbance measurements were jeopardized.

### Effect of the Orientation of 3D-TSU and Cross-Sectional Shapes on the Analytical Performance

To investigate the effect of the cross-sectional shape of the OP/AP channel of the 3D-TSU V2 device on the EF values, distinct channel shapes, including circle, triangle, square, pentagon, and obround geometries, most of which are unavailable by current micromilling and soft lithographic fabrication, were easily prototyped with stereolithographic 3D-printing by leveraging the unique fabrication opportunities of additive manufacturing. In all cases, the diameters and side lengths of the OP/AP channel were fixed to the values that ensured relatively similar volumes of the organic phase for the sake of comparability of the μ-EME data. A 2.5 mm circular cross-sectional channel was selected for the DP in all 3D-TSU devices. Whenever the 3D-TSU device was oriented in a vertical position (T, see Figure S3A) or an upside-down vertical position (upside-down T, see Figure S3B), the three phases were found to be unstable with all types of cross sections but the circular shape. However, the length of the OP plug shrank during extraction, even for the circular shape, and the extraction phases collapsed within the first 5 min of μ-EME. This observation is most likely due to gravity overcoming the surface energies of the liquid phases. In a horizontal orientation (Figure S3C), repeatable phase formation was observed throughout the μ-EME for the obround, pentagon, and circle cross-sectioned units. The triangle-shaped units exhibited poor repeatability in phase formation (RSD > 40%) when exploring the SI method, while the three-phase system was not generated at all across the square cross-sectional OP/AP channel. Hereto, a comparison of EFs determined by μ-EME was carried out across the obround, pentagon, and circular cross sections for the horizontal 3D-TSU V2 arrangement, as shown in Figure S4. Experimental extraction conditions were as follows: voltage of 250 V, time of 20 min, OP of 1-octanol (24 μL), 10 μL of AP, and 75 μL of DP (filling up the DP channel of the 3D-TSU). The pentagon cross-sectioned geometry demonstrated the highest EF (ca. 9-fold enhancement) in contrast to the obround shape with the lowest EF (ca. 5-fold enhancement). This is consistent with previous findings regarding the improved performance of devices with pentagon cross-sections in flow systems.^40^ Although the phase formation and stability of the phases were not radically different across the three cross-sectional geometries assessed, the electrical current levels recorded for the pentagon cross-section were higher than those of the other cross-section shapes in almost the first 200 s of μ-EME, thus signaling the improved mass transfer for the channel with the pentagon geometry (Figure S5).

The performance of a large-volume DP injection against low-volume μ-EME using the 3D-TSU in the mimicry of the conventional μ-EME linear configuration reported in the literature was assessed to provide invaluable insight into the potential analytical improvements (e.g., EF) harnessing the 3D-TSU arrangements.^9,18,19^Figure S6a and b demonstrate that a 3D-TSU with a DP channel of 15.5 mm length and 2.5 mm diameter using 75 μL of DP (filling the donor channel entirely) provided a ca. 5-fold EF compared to the low-volume DP injection, namely, 25 μL. This indicates that a larger volume in the 3D-TSU arrangement in a stagnant extraction mode enables enhanced electromigration and diffusion-controlled mass transfer of MB to the interface due to the increased analyte availability for μ-EME.

### Evaluation of Continuous Forward-Flow against Stop-and-Go Flow Modes of DP

In the quest to enhance the EF of μ-EME, both stopped-flow (Figure S6c−e) and continuous forward-flow (Figure S5f) approaches were implemented for the automatic SI-based handling of DP during the μ-EME process. The computer-programmable flow modes would serve the purpose of removing the electrical double layer formed between the DP and OP with the application of 250 V while replenishing the potentially depleted analyte layer at the DP/OP interface in 20 min of μ-EME.

To implement the stop-and-go flow process for the DP (300 μL), 20 min of extraction time was split into several steps:

(i) 4 steps of 300 s each (Figure S6c)
(ii) 8 steps of 150 s each (Figure S6d)
(iii) 16 steps of 75 s each (Figure S6e)

Experimental results compiled in Figure S6 indicated that there were no statistically significant differences between the EF obtained by stop-and-go flow strategies (Figure S6c–e) and that obtained by the stagnant long DP plug (Figure S6b). This might be due to the swift formation of the electrical double layer on the interface of the DP/OP in both the stagnant and the stop-and-go flow μ-EME processes. However, an 8.5-fold-enhanced EF was obtained by a continuous forward-flow of DP at 50 μL/min (Figure S6f) as compared to the stagnant short DP plug (Figure S6a), and the EF was enhanced by a factor of ca. 2 against the stagnant long DP plug (Figure S6b). It is worth noting that increasing the DP flow rate to 75 μL/min rendered a gradual washing out of the OP, therefore the narrow plug of OP left was unable to withstand the electric currents generated throughout the entire μ-EME experiment time without phase collapse.

### Bifurcation of the OP Channel for Further Enrichment

In order to increase the contact area between the DP and OP and enhance the EF of the 3D-printed μ-EME device, the OP/AP channel with a pentagon cross-section in the previous design (V2) was 3D-printed bifurcated with the same cross-sectional geometry in the V3 design (Figure 1). In this device, although the position and volume of the AP were kept the same as those of V2, the OP was distributed into two bifurcated channels. The EF capability of the bifurcated 3D-YSU V3 device bearing an AP channel in the pentagon shape (1.25 mm side) was evaluated for 3D-printed OP channels of distinct diameters. Using OP channels of 0.5, 1.25, and 1.75 mm side lengths, the EF values were ca. 2.4, 5.5, and 13.7, respectively. Although the increase of the side length rendered improved EF values, the bifurcated devices did not outperform the V2 design, notwithstanding the theoretical overall surface area of the two 1.75 mm side length channels was significantly improved against that of the V2 design. This might be associated with optical dilution effects because the larger the interface between the OP and AP is, the larger the thickness of the AP channel would be; thus, a significant mass transfer improvement would be needed to enable the same analyte concentration as that of V2 across the zone monitored by the optical fibers. It should be added that the three phases either could not be easily formed or collapsed in OP channels >1.75 mm of the bifurcated devices. However, when truncated cone-shaped pentagon OP channels were printed with a top base diameter of 1.25 mm and a bottom base diameter of 2.5 mm, the EF increased to ca. 25 under the same experimental conditions as those for V2 (Figure S7). The V3 device necessitated a larger volume of the OP (110 μL) to entirely fill up the two channels for the sake of reliable optosensing in the close vicinity of the OP/AP interface. The improvement in mass transfer is supported by higher currents detected in the V3 device compared with those recorded using the V2 device (Figure S8). To further enhance the EF, μ-EME devices with 3-fold branched and 4-fold branched OP channels were 3D-printed (Figure S9), yet reliable phase formation for the liquid plugs was not feasible in these devices. Therefore, the bifurcated OP configuration with the pentagon cross-section that enables stable three-phase formation was exploited for further method validation.

**Figure 3:**
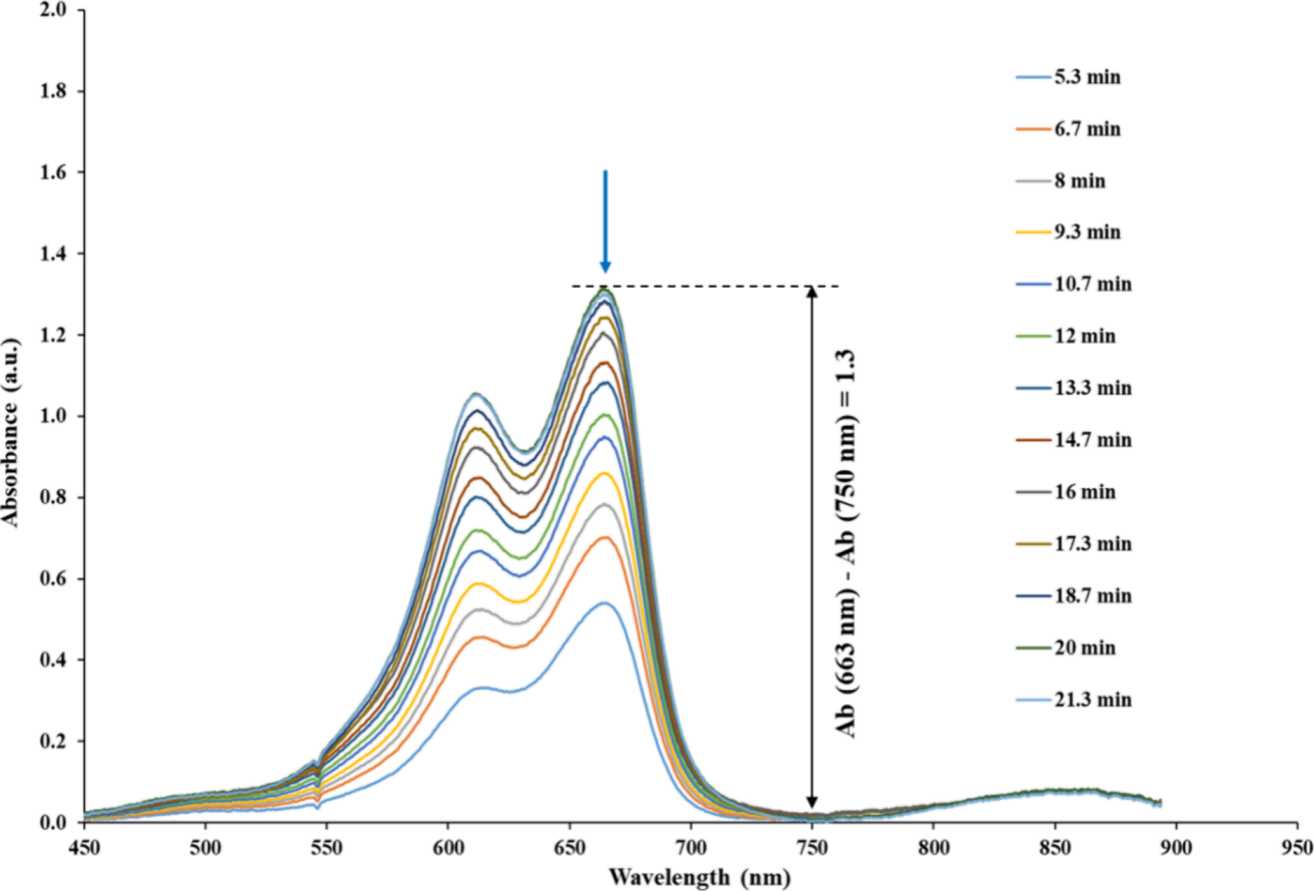
Absorbance profile of 3D-YSU V3 device with real-time recording between 5.3 and 21.3 min under a continuous flow of DP at 50 μL/min. Experimental conditions: extraction voltage, 250 V; OP, 1-octanol; AP volume, 10 μL; OP volume, 110 μL; and 1500 μL of DP (see section In-Line SI-μ-EME Procedure for more details) containing 4 mg/L of MB.

To study the effect of the extraction time on the EF, absorbance profiles were recorded in situ by optosensing between 5.3 and 21.3 min. As can be seen, the absorbance increased up to 20 min and plateaued afterward (Figure 3). Therefore, the extraction time was set to 20 min for the subsequent validation steps.

## Method Validation

The figures of merit of the automatic SI-3D-YSU-μ-EME optosensing method for MB determination were as follows: (i) linear range, (ii) limit of detection (LOD), (iii) limit of quantification (LOQ), (iv) intra- and inter-day repeatability, and (v) reusability of the 3D-YSU. The linear dynamic range spanned from 0.05 to 2.0 mg/L with an *R*^2^ of 0.994 (*Y* = 0.394[MB (mg/L)] – 0.0093) and sensitivity of 0.394 a.u. ×L/mg according to IUPAC guidelines (Figure S10). LOD and LOQ were 0.015 and 0.05 mg/L, respectively, as calculated based on the S/N = 3 and S/N = 10 criteria. Intra- and interday RSD% values were determined to be 10% and 16% (*n* = 3, 0.25 mg/L), respectively, using, in all cases, fresh 3D-YSUs. To assess the reusability of the 3D-printed devices, the SI setup was programmed to perform μ-EME seamlessly with the same device for an MB concentration of 0.25 mg/L. Experimental results indicated that a single device could be reused up to 9 times with an RSD of 10.3% (Table S2). After 9 reuses, a significant drop in the absorbance signals of MB was observed. The entire SI-3D-YSU-μ-EME protocol, including conditioning of the SI system, MB microextraction, optosensing, and regeneration of the 3D-YSU, took ca. 35 min per run.

The greenness of the 3D-YSU-μ-EME method was assessed through the AGREE approach.^41^ The inputs of the 12 criteria in AGREE (see Figure S11) are summarized below: (1) online, (2) 1050 μL of wastewater, (3) off-line (lab) experiments in this proof-of-concept work, (4) three or fewer steps as the adjustments were made solely to the sample’s ionic strength, (5) automatic and miniaturized, (6) no derivatization, (7) approximately 12.8 mL and/or g of waste, (8) a throughput of ca. 1.7 runs per hour with MB as the exclusive target analyte, (9) UV–vis spectrometry, (10) some reagents are biobased, (11) 10.18 mL and/or g, and (12) “highly flammable” and “toxic to aquatic life” due to the use of 1-octanol. The AGREE score of the developed method for MB determination was 0.59, and thus it offers superior environmental friendliness as compared to previously reported spectroscopic and chromatographic methods with green scores spanning from 0.26 to 0.55.^42−48^

As evinced in a comprehensive review by Hansen et al.^14^ on micro/millifluidic platforms accommodating supported EME, a large number of studies using flow-based EME platforms required prolonged extraction times to yield EF < 20, e.g., 33 min for the determination of biogenic amines^22^ and 45 min for the determination of the amitriptyline and its metabolites.^49^ Additional offline detection procedures contribute significantly to their total analysis time.^14^ It should be noted that the previous articles in the literature primarily focused on SLM-based EME exploiting PMMA micromachined devices for the extraction of acidic and basic drugs.^14^ In those articles, throughput has been barely reported, most likely due to the offline detection step and the lack of reusability of SLMs. In fact, reusability has been only discussed in one study, in which the membrane should be replaced after 6 runs and a new multilayer microfluidic platform had to be fabricated by laser cutting after 30 experiments.^24^

To evaluate the real-life applicability and the trueness of the automatic SI-3D-YSU-μ-EME optosensing method, the amount of MB was determined in synthetic textile dye and urban wastewater samples. MB is widely applied in industrial processes to dye silk, wood, paper, leather, plastics, and cotton.^50,51^ In fact, the complete removal of MB by wastewater treatment plants (WWTP) is particularly difficult due to its high water solubility. MB released into the environment through waste effluents of such dying industries can limit sunlight penetration in water and reduce photosynthetic activity and dissolved oxygen concentration, which might have adverse effects on the ecosystem. Consequently, determining the amounts of MB in industrial wastewater and environmental water is of utmost relevance, especially in the evaluation of the efficiency of chemical remediation procedures based on, e.g., activated carbon, silica, clay, industrial solid wastes, and biomass (bio)sorbents in WWTP.^51^

As indicated above (see section In-Line SI-μ-EME Procedure), the conductivity of all wastewater samples and standards for matrix-matched calibration was kept constant to that equivalent to 25 mmol/L sodium chloride. The relative recoveries (RR%) in wastewater samples ranged from 88% to 91%, with RSD% values (*n* = 3) in the range of 11–19% (see Table S3), indicating the ruggedness of the in-line microextraction and optosensing detection procedures and the inexistence of significant multiplicative matrix interference after ionic strength adjustment.

## Conclusion

This study was aimed at leveraging the unique opportunities offered by stereolithographic 3D-printing technology to address the low enrichment capabilities encountered by the standard nonsupported μ-EME systems using polymeric tubing or pipet tips. The herein prototyped nonsupported 3D-printed devices offered substantial advantages in terms of accommodating larger volumes of DP in contrast to their conventional low-volume single-channel μ-EME counterparts. Compared to our previous research work^38^ in which a 3D-printed unit was exploited using static low microliter volumes of sample to enhance the extraction recovery against those of standard tubing but with EF < 1, the herein proposed μ-EME-3D-TSU/YSUs integrated into a fully automatic SI system enabled unsupervised stop-and-go and forward-flow movement of the DP, which helped eliminate the electrical double layer formed between the DP and OP while renewing the analyte-depleted layer in contact with the OP. Additionally, bifurcation of the OP channel enhanced the contact area between the DP and OP, and direct optosensing of the AP prevented dilution of the enriched plug toward the detector, thereby further ameliorating the EF of the μ-EME method. As a proof-of-concept of its applicability, the automatic SI-3D-YSU-μ-EME optosensing system was harnessed to determine the amount of MB in high matrix samples, including synthetic textile dye and urban wastewaters. Current work is underway in our laboratory to further exploit the flexibility of the automatic 3D-YSU-based microextraction system with an improved EF as a front end to liquid chromatography for the determination of environmentally relevant organic pollutants and metabolites thereof.

## Supporting Information Available

Details (i) the reagents and chemicals; (ii) the flow system and instrumentation; (iii) the operational procedure for automatic μ-EME; (iv) the fabrication, design and images of the 3D printed fluidic structures with distinct configurations and orientations; (v) the analysis of real samples; (vi) the experimental data of optimization parameters and exploration of V3 against V2 prototypes; (vii) the electric currents recorded throughout in-line μ-EME of distinct devices; and (viii) quantitative data for greenness evaluation using the AGREE tool (PDF)
