# Supplementary material for: Enhancing the Concentration Capability of Nonsupported Electrically Driven Liquid-Phase Microextraction through Programmable Flow Using an All-In-One 3D-Printed Optosensor: A Proof of Concept
Source: Anal Chem. 2024 Jun 25;96(27):11068–75. doi: 10.1021/acs.analchem.4c02139 (PMC11238157; doi:10.1021/acs.analchem.4c02139)
Supplement: Supplementary file 1 — ac4c02139_si_001.pdf [file ac4c02139_si_001.pdf]

Supporting Information

Enhancing the Concentration Capability of Nonsupported Electrically Driven Liquid-Phase Microextraction through Programmable Flow Using an All-In-One 3D-Printed Optosensor: A Proof of Concept

Ali Sahragard[*a], Enrique Javier Carrasco-Correa[b], David J Cocovi-Solberg[c], Pavel Kubáň[d], Manuel Miró[*a]

[a]FI-TRACE Group, Department of Chemistry, Faculty of Science, University of the Balearic Islands, Carretera de Valldemossa km 7.5, E-07122 Palma de Mallorca, Illes Balears, Spain.

[b]CLECEM Group, Department of Analytical Chemistry, University of Valencia, C/ Doctor Moliner, 50, 46100, Burjassot, Valencia, Spain.

[c]University of Natural Resources and Life Sciences, Vienna, Department of Chemistry, Institute of Analytical Chemistry, Muthgasse 18, 1190, Vienna, Austria

[d]Institute of Analytical Chemistry of the Czech Academy of Sciences, Veveří 97, CZ-60200 Brno, Czech Republic.

21 pages

11 figures

3 Tables

*Corresponding authors. E-mails: ali.sahragard@uib.es, manuel.miro@uib.es

Table of Content

Chemical and standards……………………………………………………………...S3

Fabrication of the millifluidic units…………………………………………………..S3

Flow system and optical detection…………………………………………………...S4

µ-EME instrumentation……………………………………………………. ……. S5

Real Samples………………………………………………………………………... S6

EF calculation……………………………………………………………………..... S6

Figure S1……………………………………………………………………………. S7

Figure S2……………………………………………………………………………. S8

Figure S3……………………………………………………………………………. S9

Figure S4…………………………………………………………………………... S10

Figure S5…………………………………………………………………………... S11

Figure S6…………………………………………………………………………... S12

Figure S7…………………………………………………………………………... S13

Figure S8…………………………………………………………………………... S14

Figure S9…………………………………………………………………………... S15

Figure S10……………………………………………………………...…....... S16

Figure S11…………………………………………………………….……... S17

Table S1…………………………………………………………………………… S18

Table S2…………………………………………………………………………… S19

Table S3…………………………………………………………………………… S20

Reference………………………………………………………………………….. S21

Chemicals and standards. Sodium hydroxide, sodium chloride (NaCl), 1-octanol, ethanol, 2-isopropanol (IPA), MB, and sodium carbonate were acquired from Fisher Scientific (Madrid, Spain). A 250 mg/L MB stock solution in deionized (DI) water was serially diluted with DI to prepare working standard solutions. Additionally, an NaCl solution (2 mol/L) prepared in DI was used to adjust the ionic strength of the standards used for the external matrix-matched calibration and the wastewater samples to that equivalent to 25 mmol/L NaCl.

Fabrication of the millifluidic units. FreeCAD software (www.freecadweb.org) was employed to design the 3D-TSU/YSU devices. The 3D models were converted into computer-aided design (CAD) files (STL), and imported into the Preform software (Formlabs, Somerville, USA) for designing of replicates, positioning of the objects, and slicing. The resulting computer-aided manufacturing (CAM) file (FORM) was submitted to the stereolithographic Form 3 printer (Formlabs) and the units were 3D-printed with an adaptive resolution using the FLGPCL02 (Formlabs) clear resin with the incorporation of minisupports of 4 mm height. Upon completion of the 3D printing process, the 3D-TSU/YSUs were retrieved from the moving platform and immersed in IPA for 10 min, and all channels were flushed with IPA using medical syringes to remove remnants of non-polymerized liquid resin. Then, the units underwent a three-step washing protocol (15 min each) using ultrasonic cleaning with IPA, DI, and IPA again, respectively. Subsequently, the 3D-TSU/YSUs were dried by an N_2 stream through the channels. To finalize the printing process, the units underwent a one-hour exposure to UV light (5000 W) using the APPLIGENE system, the CL-1000 model from Analytik Jena US LLC (Upland, Canada). Various threads were then manually tapered, and units were kept in the dark to preserve the stiffness of the prints.

Flow system and optical detection. A sequential injection (SI) apparatus served as the millifluidic platform for automatic µ-EME and optosensing detection. The setup of the SI included a bidirectional microsyringe pump (SP) with a 30 mm stroke, featuring a 1000 µL borosilicate glass syringe from Cavro Scientific Instruments (San Jose, CA, USA), and a 10-port multi-position selection valve (MPV, VICI Cheminert 11X-0231L) purchased from Valco Instruments Co. Inc (Schenkon, Switzerland). The SP housed a head valve (HV) equipped with three ports (In, Up, and Out) that was employed to select the carrier (water, In position) or samples/reagents (Out position). Avantor (Llinars del Vallès, Spain) supplied all polytetrafluoroethylene (PTFE) tubing utilized for connections. The HV enabled communication between the SP and MPV via the Out position, employing a ca. 35-cm long holding coil (HC1) made of PTFE tubing with 1.5 mm ID and 2.4 mm OD. The CocoSoft 7.1 freeware was exploited for an unsupervised operation of SP, HV, and MPV and automatic control of the overall hydrodynamic variables. The in-line downstream spectrophotometric detection for V1 has been described elsewhere.[1] In brief, a USB4000 UV-vis spectrometer (Ocean Optics, Largo, Florida, USA) was linked through optical fibers (specifically QP400-1-UV-vis, from Ocean Optics) to an ISS UV-vis integrated sampling system (also from Ocean Optics) for in-line spectrophotometric detection of V1-derived AP. This system is composed of a UV-vis light source, an integrated cell holder, and a flow-through quartz cell (model 178-712-QS, with a path length of 10 mm and a volume of 18 µL, purchased from Hellma GmbH, Müllheim, Germany). For the detection, AP was first retrieved from the OP/AP channel of the V1 device to HC1 (see **Figure 2**) and then injected into the flow cell connected to port #2 (not shown in **Figure 2**).

Optosensing detection directly in the V2 and V3 devices was accomplished by a halogen light source (Halogen Light Source HL-2000, OceanOptics) and the USB4000

UV-vis spectrometer operating as the light source and the detector, respectively. The optical fibers (P50-1-UV-VIS, with a core size of 50 µm, connected to the light source, and QP400-1-UV-VIS, with a core size of 400 µm, connected to the detector, both from Ocean Optics) were manually tightened by press-fitting to ca. 1.5 mm depth into the holes of the 3D-TSU/YSU as detailed in section **Fabrication of the millifluidic units** (see also label H in **Figure S1II**). The SpectraSuite software (version 1.6.0.11, Ocean Optics) enabled data recording and setting the optical detection parameters, that is, an integration time of 100 ms, an average of 10 scans, and a Boxcar width of 2. For the full spectra recording within the range of 178-800 nm, the CocoSoft 7.1 freeware was programmed to (i) open the SpectraSuite software for the USB spectrometer, (ii) click on "convert to overlay" on the right-hand side of the main page of the SpectraSuite software, and (iii) return to CocoSoft freeware. Subsequently, Excel macros were employed to process the spectral data, using an analytical wavelength of 663 nm and a reference wavelength of 750 nm throughout.

µ-EME instrumentation. Two (in V1 and V2) and three (in V3) 16 mm-long and 0.25 mm-thick tubular platinum wires (99.9%, Thermo Scientific, Madrid, Spain) served as µ-EME electrodes. The platinum electrodes were fixed in pipette tips with liquid resin (FLGPCL02, Formlabs) by UV polymerization, and positioned in the 3D-TSU/YSUs via pre-embedded holes (see label B and F in **Figure S1** and **Figure 1**). A 0-300 V power supply (ES 0300-0.45, Delta Elektronika, Zierikzee, The Netherlands) was utilized for an unsupervised application of an electrical potential. Concurrently, a UT70B electronic multimeter (TENMA, Spain) was used for real-time recording of the current profiles.

Real samples. Two 24 h-pooled influent wastewater samples (# 1: April 2nd-3rd, 2023 and # 2: April 13th-14th 2023) were collected from a wastewater treatment plant that receives urban and light industrial effluents from the city of Palma (EDAR-2, Illes Balears, Spain) with a load of 564,000 population-equivalent. Wastewater # 1 contained 15 mg/L total P, 88 mg/L N, 2768 mg/L COD (chemical oxygen demand), and 550 mg/L BOD_5 (biochemical oxygen demand) while wastewater # 2 contained 16 mg/L total P, 90 mg/L N, 3272 mg/L COD, and 530 mg/L BOD_5. Additionally, a synthetic textile dyeing surrogate wastewater was prepared to contain 25 mmol/L NaCl and 150 mg/L Na_2CO_3 as recommended elsewhere.[1] The conductivity of all real samples was adjusted with 2 mol/L NaCl to match that of 25 mmol/L solution of NaCl (2361 µS/cm) at room temperature (25° C) to avoid salt-depending µ-EME results. Conductometric measurements were performed using a COND 7+ conductometer with COND Cell mod 2301 T (XS Instruments, Carpi, Italy). A conductometric calibration curve (Y = 93.644 X + 19.87; R^2 = 0.9998) was established using 1-30 mmol/L NaCl solutions. The original conductivities of urban wastewater samples # 1 and # 2 were found to be 2280 and 1236 µS/cm at 25 °C, respectively.

EF calculation. EF was calculated by dividing the absorbance of the AP after µ-EME by the absorbance of the DP before µ-EME, both with the same volume according to equation 1:

$$EF = A_{AP\ after\ \mu\text{-}EME}/A_{DP\ before\ \mu\text{-}EME} \qquad \text{(Equation1)}$$

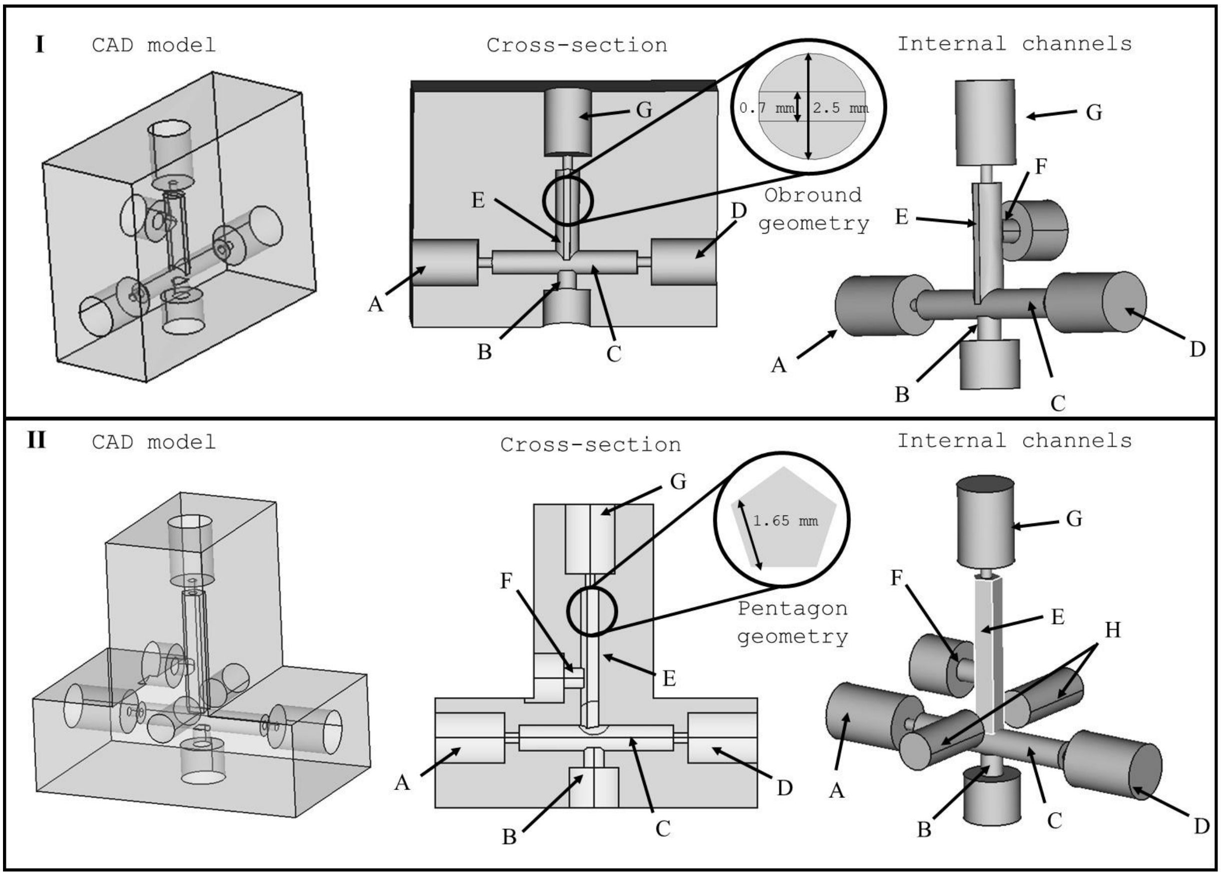

Figure S1. Designs of two flow-through 3D-TSU devices with obround (V1) and pentagon (V2) cross-section for AP that are exploited for the dynamic µ-EME of MB. **I**) With downstream optical detection (V1) and **II**) with direct optosensing in the vicinity of the OP/AP interface (V2). A: thread for the DP input, B: hole for the positive electrode in the DP channel, C: DP channel, D: thread for the DP output (waste), E: OP/AP channel, F: hole for the negative electrode in the AP channel, G: thread for the OP/AP input; H: holes for the optical fibers.

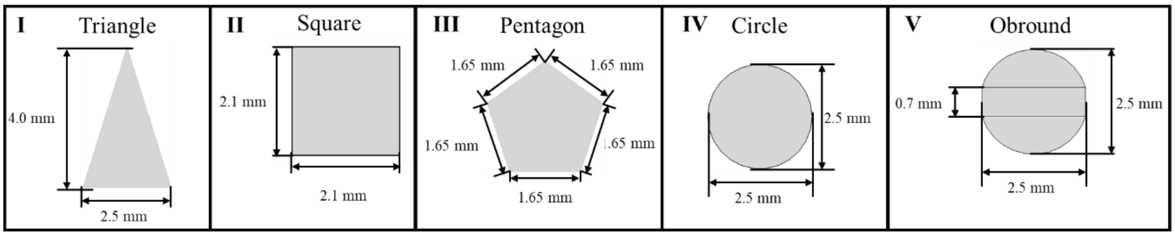

Figure S2. Distinct cross-section geometries of the AP/OP channel explored for the 3D-TSU V2 optosensing devices.

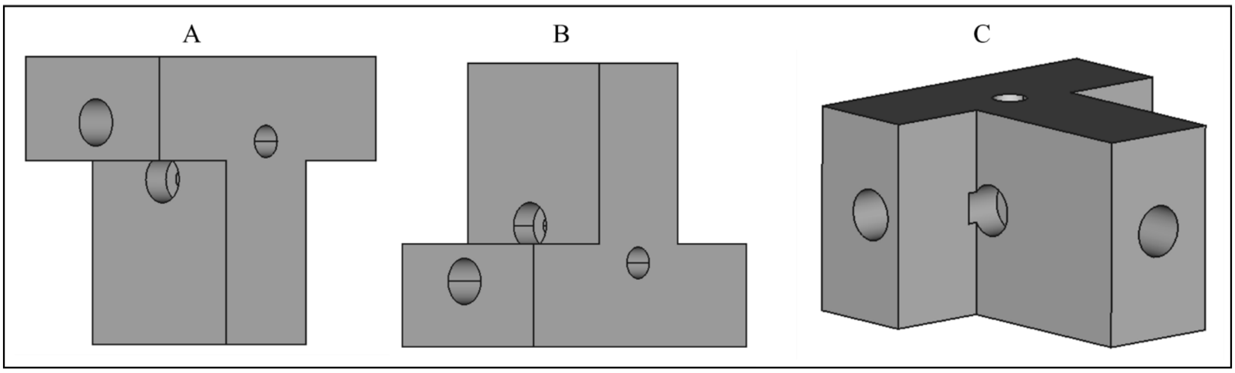

Figure S3. Distinct spatial disposition of the 3D-TSU V2 optosensing devices: A) T-shaped, B) upside down T-shaped, and C) horizontal T-shaped.

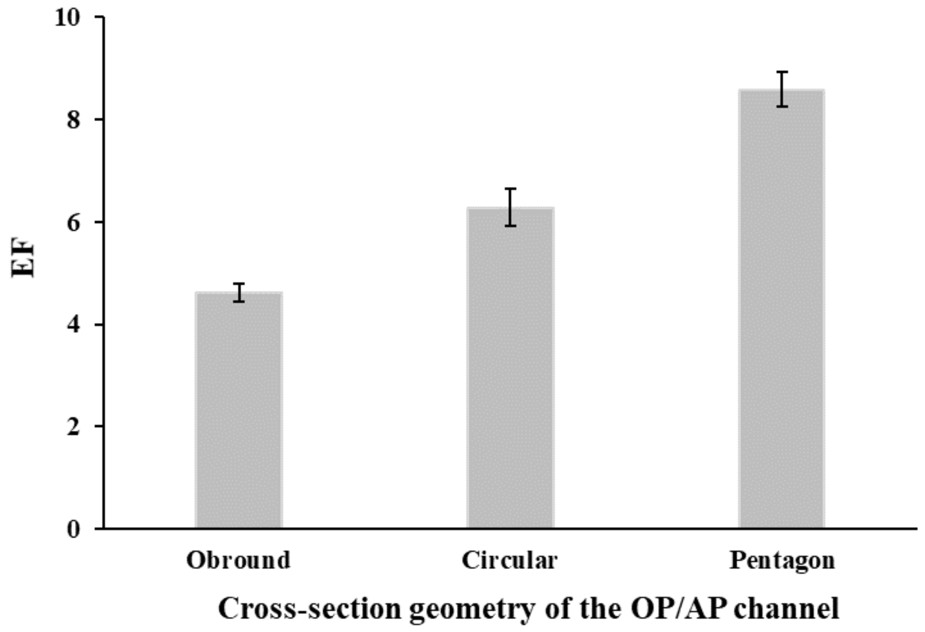

Figure S4. EF values obtained with various OP and AP cross-sectioned channels for the 3D-TSU V2 device in a stagnant DP extraction format. Experimental conditions: extraction voltage, 250 V; extraction time, 20 min; OP, 1-octanol; OP volume, 24 µL; AP volume, 10 µL; DP volume, 75 µL containing 4 mg/L of MB.

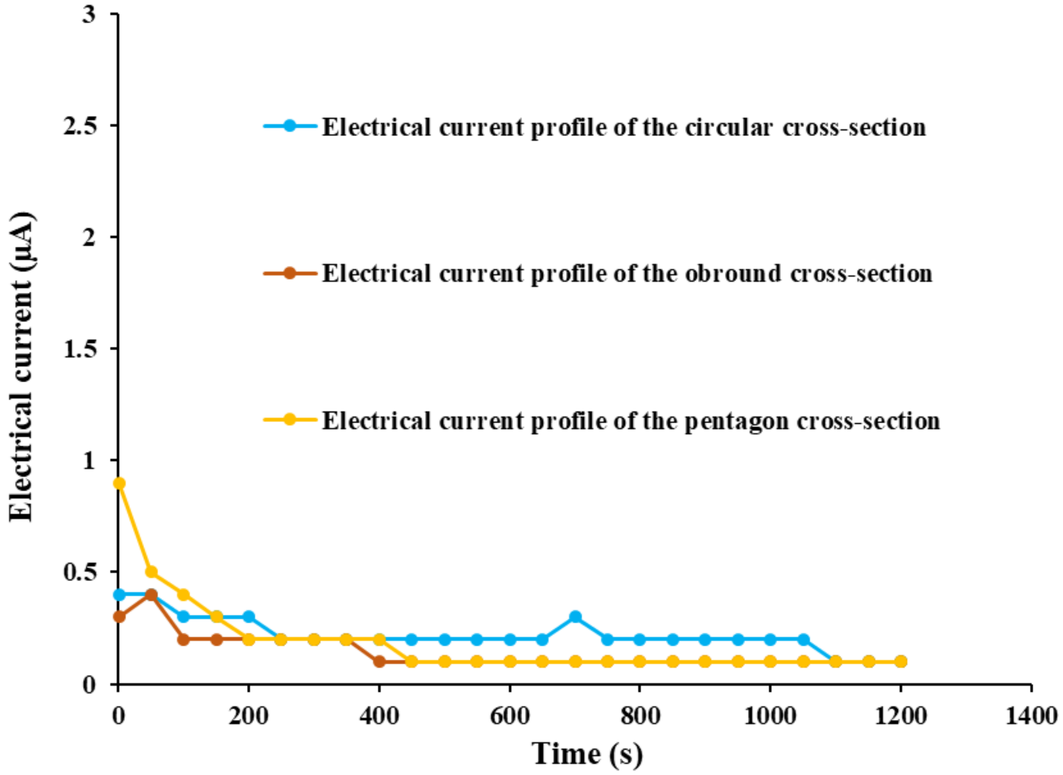

Figure S5. Electrical current profiles recorded in the time course of the SI-3D-TSU-µ-EME using different cross-sectional geometries for the OP and AP channels. Extraction conditions: extraction voltage, 250 V; extraction time, 20 min; OP, 1-octanol; OP volume, 24 µL; AP volume, 10 µL; DP volume, 1050 µL containing 4 mg/L of MB.

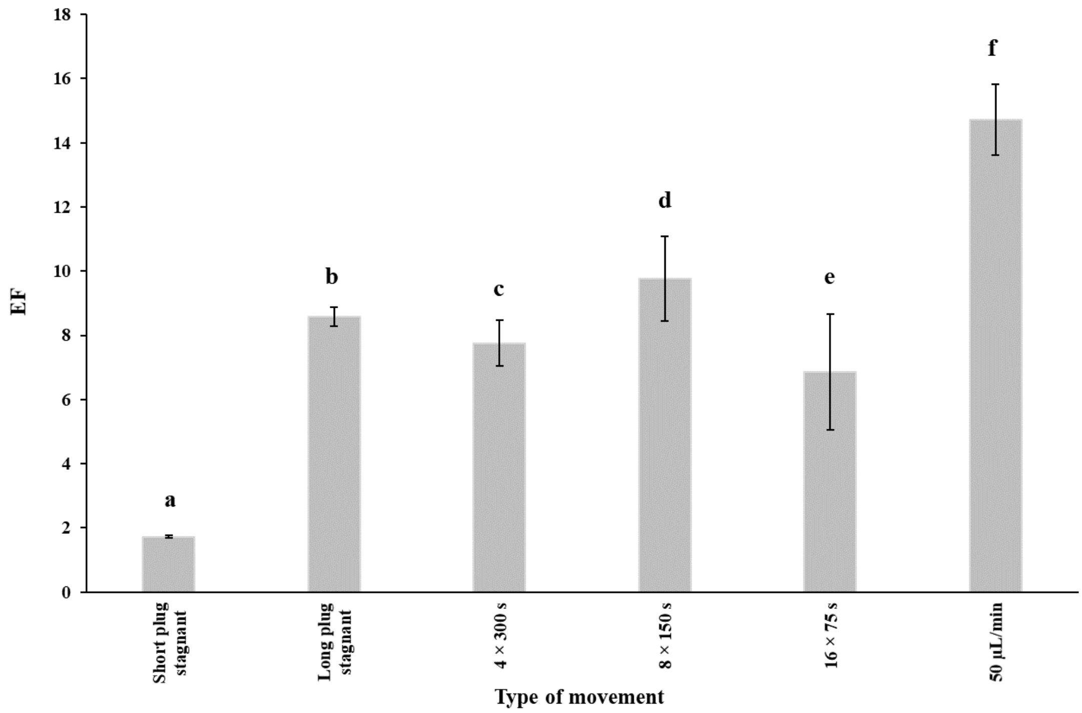

Figure S6. EF values using stagnant and various dynamic DP flow modes (stop-and-go and continuous) for the detection of 4 mg/L of MB. Experimental condition: extraction voltage, 250 V; extraction time, 20 min; OP, 1-octanol; AP volume, 10 µL; OP volume, 24 µL; and DP volumes, 25 (a), 75 (b), 300 (c), 300 (d), 300 (e), and 1050 µL (f).

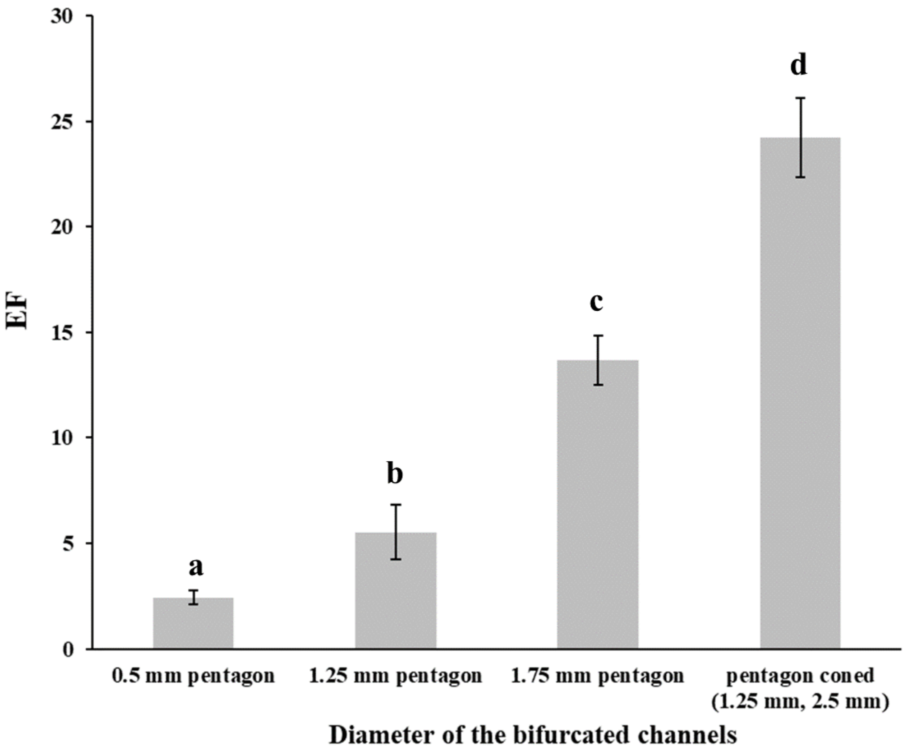

Figure S7. EF values using various bifurcated 3D-YSU devices with different pentagon side lengths for the detection of 4 mg/L of MB. Experimental condition: extraction voltage, 250 V; extraction time, 20 min; OP, 1-octanol; AP volume, 10 µL; OP volume, (a) 25, (b) 40, (c) 60, and (d) 110 µL; DP volume, 1050 µL; and flow rate, 50 µL/min.

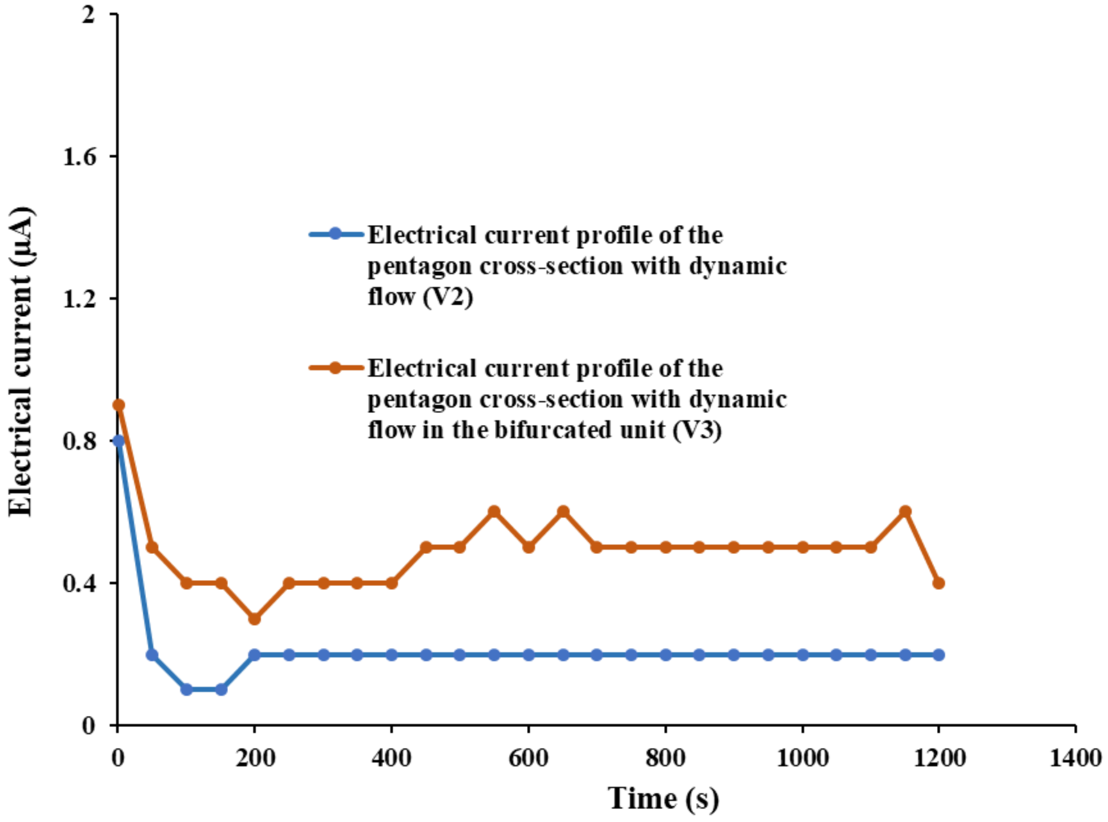

Figure S8. Electrical current profiles as obtained by SI-3D-TSU/YSU-µ-EME exploiting V2 and V3 devices. Extraction conditions: extraction voltage, 250 V; extraction time, 20 min; OP, 1-octanol; OP volumes, 24 µL for V2 and 110 µL for V3 devices; AP volume, 10 µL; DP volume, 1050 µL containing 4 mg/L of MB; and flow rate, 50 µL/min.

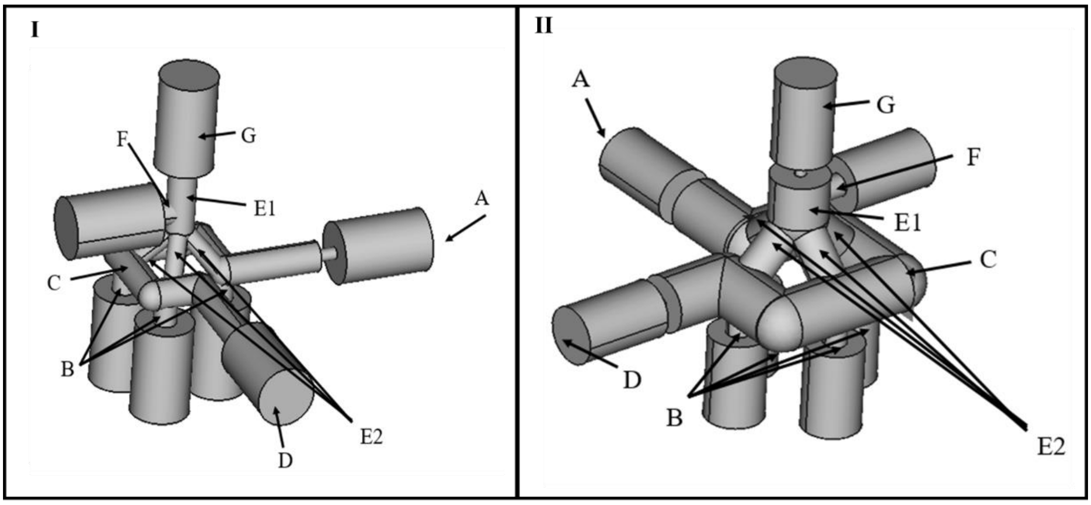

Figure S9. Design of the 3D-printed optosensing device with **I**) trifurcate (threefold branched) and **II**) quadrifurcate (fourfold branched) OP channel (E2) for the dynamic µ-EME of MB. The labels are identical to the designs in Figure 1 and Figure S1, yet three and four holes for the electrodes that are tapered in the DP channel (C) were designed in I and II, respectively. The AP channel is labelled as E1 and the OP channels are labelled as E2.

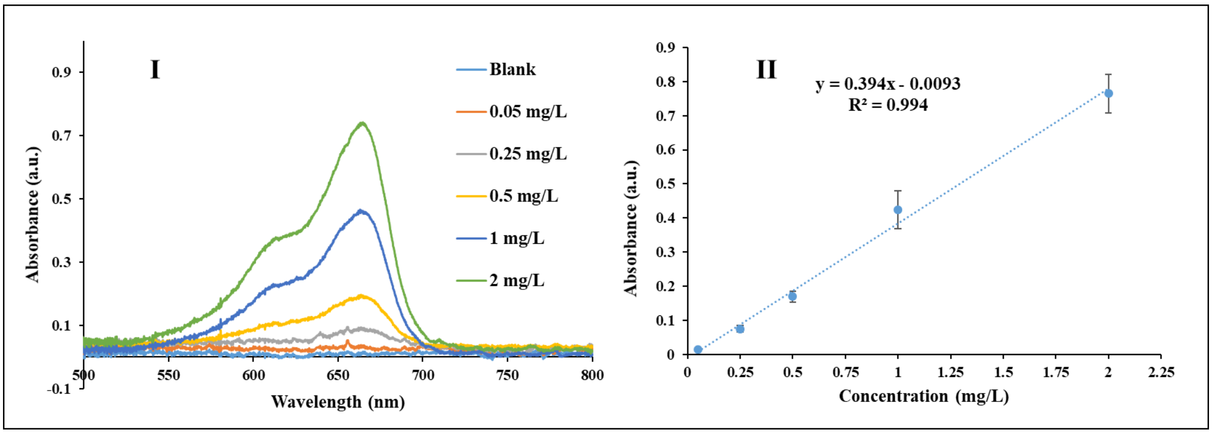

Figure S10. (I) UV-vis spectra obtained at different concentrations of MB via SI-3D-TSU/YSU-µ-EME and (II) Calibration curve with a linear dynamic range spanning from 0.05 to 2 mg/L. Extraction conditions: extraction voltage, 250 V; extraction time, 20 min; OP, 1-octanol; OP volume, 110 µL; AP volume, 10 µL; DP volume, 1050 µL; and flow rate, 50 µL/min.

Analytical Greenness report sheet

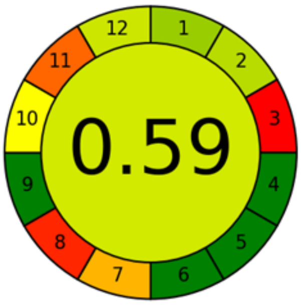

1. Sample treatment
2. Sample amount
3. Device positioning
4. Sample prep. stages
5. Automation, miniaturization
6. Derivatization
7. Waste
8. Analysis throughput
9. Energy consumption
10. Source of reagents
11. Toxicity
12. Operator's safety

Criteria	Score	Weight
1. Direct analytical techniques should be applied to avoid sample treatment.	0.7	2
2. Minimal sample size and minimal number of samples are goals.	0.64	2
3. If possible, measurements should be performed in situ.	0.0	2
4. Integration of analytical processes and operations saves energy and reduces the use of reagents.	1.0	2
5. Automated and miniaturized methods should be selected.	1.0	2
6. Derivatization should be avoided.	1.0	2
7. Generation of a large volume of analytical waste should be avoided, and proper management of analytical waste should be provided.	0.35	2
8. Multi-analyte or multi-parameter methods are preferred versus methods using one analyte at a time.	0.08	2
9. The use of energy should be minimized.	1.0	2
10. Reagents obtained from renewable sources should be preferred.	0.5	2
11. Toxic reagents should be eliminated or replaced.	0.2	2
12. Operator's safety should be increased.	0.6	2

Figure S11. Green metrics for the SI-3D-YSU-μ-EME optosensing method for MB determination using AGREE tool.

Table S1. Summary of the operational procedure for the automatic SI-µ-EME-UV-vis optosensing detection of MB using bifurcated 3D-YSU.

Dispensing DP into the PTFE tubing connected to the donor channel of the 3D-YSU (1050 µL)

⇩

Dispensing OP and AP into the OP/AP channel of 3D-YSU (110 µL OP and 10 µL AP)

⇩

Dispensing DP into the Y-shaped platform for stabilization of the DP/OP interface (50 µL)

⇩

Starting the µ-EME (250 V)

⇩

Applying continuous flow of the DP for 20 min with a flow rate of 50 µL/min

⇩

Recording the spectra using the three click strategy (Analytical wavelength of 663 nm and reference wavelength of 750 nm)

⇩

Washing the 3D-YSU channels by 300 µL ethanol, 500 µL water, and 2050 µL air

Table S2. Investigation of the reusability of a 3D-YSU-μ-EME platform using a nominal concentration of 0.25 mg/L MB.

**Run. No**	**Absorbance (a.u.)**	**Concentration found (mg/L)**	**Average of concentrations (mg/L) (n=9)**	**SD**	**RSD%**
1	0.12	0.33	0.29	0.03	10.3
2	0.11	0.30			
3	0.11	0.30			
4	0.11	0.30			
5	0.10	0.28			
6	0.11	0.30			
7	0.09	0.25			
8	0.10	0.28			
9	0.08	0.23			
10	*0.04*	*0.13*			

Table S3. Determination of MB in synthetic textile dyeing and urban wastewaters.

Sample	Spiked conc. (mg/L)	Found conc. (mg/L)	RR%	RSD%	Bias%
**Synthetic textile wastewater**	0	ND	-	-	
	1.0	0.91	91	18	-9
**Urban wastewater 1**	0	ND	-	-	
	1.0	0.9	90	19	-10
**Urban wastewater 2**	0	ND	-	-	
	1.0	0.88	88	11	-12

ND: non-detected.

REFERENCE

(1) Sahragard, A.; Dvořák, M.; Pagan-Galbarro, C.; Carrasco-Correa, E. J.; Kubáň, P.; Miró, M. 3D-printed stereolithographic fluidic devices for automatic nonsupported microelectromembrane extraction and clean-up of wastewater samples. *Anal. Chim. Acta* **2024**, *1297*, 342362. DOI: 10.1016/j.aca.2024.342362.
